# Production of Esters in *Escherichia coli* Using Citrate Synthase Variants

**DOI:** 10.3390/microorganisms12071338

**Published:** 2024-06-29

**Authors:** Jacoby C. Shipmon, Pasupathi Rathinasabapathi, Michael L. Broich, Hemansi, Mark A. Eiteman

**Affiliations:** 1 School of Chemical, Materials and Biomedical Engineering, University of Georgia, Athens, GA 30602, USA; jacobyship@gmail.com (J.C.S.);; 2 Department of Genetic Engineering, SRM Institute of Science and Technology, Chengalpattu District, Kattankulathur 603202, Tamil Nadu, India; 3 Department of Microbiology, Central University of Haryana, Mahendergarh 123029, Haryana, India

**Keywords:** acetate esters, citrate synthase, propyl acetate, point mutation, batch fermentation

## Abstract

Acetate esters comprise a wide range of products including fragrances and industrial solvents. Biosynthesis of esters offers a promising alternative to chemical synthesis because such routes use renewable carbohydrate resources and minimize the generation of waste. One biochemical method for ester formation relies on the *ATF1* gene from *Saccharomyces cerevisiae*, which encodes alcohol-O-acyltransferase (AAT) which converts acetyl-CoA and an exogenously supplied alcohol into the ester. In this study, the formation of several acetate esters via AAT was examined in *Escherichia coli* chromosomally expressing citrate synthase variants, which create a metabolic bottleneck at acetyl-CoA. In shake flask cultures, variant strains generated more acetate esters than the strains expressing the wild-type citrate synthase. In a controlled bioreactor, *E. coli* GltA[A267T] generated 3.9 g propyl acetate in 13 h, corresponding to a yield of 0.155 g propyl acetate/g glucose, which is 18% greater than that obtained by the wild-type GltA control. These results demonstrate the ability of citrate synthase variants to redistribute carbon from central metabolism into acetyl-CoA-derived biochemicals.

## 1. Introduction

Acetate esters have many applications in the food and chemical industry. They are naturally produced by yeast such as *Saccharomyces cerevisiae*, and, at low concentrations, create the unique taste and odor of fermented beverages [1]. Important acetate esters for flavoring include ethyl acetate (fruity, solvent aroma), isobutyl acetate (sweet, fruit), isoamyl acetate (banana) and 2-phenylethyl acetate (rose) [2]. Several esters are also industrial solvents due to their biodegradability and low toxicity [3]. Biosynthesis of esters offers a promising alternative to chemical synthesis because such routes use renewable carbohydrate resources, and because microorganisms can perform conversions at ambient conditions [4]. The common pathways for ester biosynthesis are mediated by (i) esterases, (ii) hemiacetal dehydrogenases, (iii) Baeyer–Villiger monooxygenases and (iv) alcohol-O-acyltransferases (AATs). The reactions catalyzed by AATs and esterases are redox neutral, whereas Baeyer–Villiger monooxygenases and hemiacetal hydrogenases, respectively, require NAD(P)H and NAD(P) [5].

The absence of cofactors in a biosynthetic reaction provides an advantage in metabolic engineering since a production microbe does not need to be engineered to alter or redirect redox equivalents. Esterases, including lipases, are ubiquitous enzymes that typically catalyze the hydrolysis of ester bonds, resulting in the formation of an alcohol and a carboxylic acid such as acetate. Under aqueous conditions, ester formation via esterases is thermodynamically unfavorable [6]. Hence, industrial production of esters via esterases is typically performed in organic solvents or high substrate concentrations [5]. AATs mediate ester biosynthesis via the transfer of an acyl group from an acyl-CoA such as acetyl-CoA to an alcohol. Because thioester bonds are energy rich, the transfer of the acetyl moiety from acetyl-CoA to an acceptor alcohol results in a negative ΔG°. AATs vary in alcohol and acyl-CoA specificities [5]. The widely studied *S. cerevisiae* alcohol-O-acetyltransferases (EC 2.3.1.84) Atf1 and Atf2 synthesize a range of acetate esters from acetyl-CoA [7].

Several previous studies have used metabolic engineering with Atf to examine ester formation. One approach involves introducing a free alcohol into the medium, which then will transport into a cell expressing Atf and react with intracellular acetyl-CoA to yield the corresponding acetate ester. For example, addition of 2 g/L propanol led to 800 mg/L propyl acetate in shake flasks [8]. Similarly, inclusion of 880 mg/L isoamyl alcohol into an anaerobic culture of cells led to 230 mg/L isoamyl acetate [9]. Another approach is to have the Atf-expressing host also generate a biochemically tractable alcohol. For example, expressing the 2-ketoisovalerate pathway for isobutanol formation, *E. coli* generated 17 g/L isobutyl acetate in a shake flask culture using hexadecane as an in situ extractant [10], and 36 g/L isobutyl acetate in a batch bioreactor using three sequential off-gas water receivers to collect the volatile ester [11]. Similarly, incorporation of the butanol pathway from *Clostridium acetobutylicum* into *E. coli* expressing Atf, and capturing the volatile ester in water receivers, generated 22.8 g/L isobutyl acetate [12].

Because Atf1 mediates multiple reactions of short- to medium-length alcohols with acetyl-CoA, kinetic parameters for this enzyme have been reported for each particular alcohol/ester combination. For example, the K_M(app)_ for acetyl-CoA in ethyl acetate formation is 45 μM, and the K_M(app)_ for acetyl-CoA in isoamyl acetate synthesis is 25 μM. In contrast, the K_M(app)_ for isoamyl alcohol is 25 mM [13]. Although the K_M_ for alcohols is often greater, suggesting this substrate could be the rate-limiting substrate during native yeast fermentation [14], the intracellular concentration of acetyl-CoA in wild-type *Escherichia coli* ranges between 20 and 600 μM [15], indicating that, for most naturally occurring esterification reactions, particularly those in which the alcohol is exogenously supplied, acetyl-CoA is the limiting substrate. Indeed, acetyl-CoA/CoA levels directly affect acetate esters in *S. cerevisiae* [16]. Thus, the intracellular availability of acetyl-CoA is likely a critical factor in metabolic engineering strategies for the biosynthesis of acetate esters using Atf1.

Acetyl-CoA occupies a central position in metabolism, and serves as a precursor of anabolic reactions, as an allosteric regulator of enzymatic activities and as a key determinant of protein acetylation. Many strategies have been developed to increase the flux to acetyl-CoA [17]. During aerobic steady-state growth of *E. coli* on glucose, 62% of the acetyl-CoA flows through citrate synthase [18]. In the production of esters via AATs, citrate synthase (coded by the *gltA* gene) is the primary biochemical competitor for the mutual substrate acetyl-CoA. Although a *gltA* knockout increases acetyl-CoA availability for formation of several acetyl-CoA-derived products, including citramalate [19] and mevalonate [20], a Δ*gltA* strain does not grow on glucose as the sole carbon source, and the medium requires supplementation with glutamate for growth. An alternative to the deletion of citrate synthase is a reduction in the expression or the activity of this enzyme. Previously, the formation of acetate, derived as an overflow metabolite from acetyl-CoA, was correlated to reduced activity of citrate synthase engineered by targeted amino acid substitutions in the enzyme [21], with some strains achieving acetate yields greater than 0.24 g/g compared to about 0.05 g/g in the wild-type citrate synthase control. This approach has also been used for the formation of citramalate [22] and 3-hydroxybutyrate [23]. Substitutions in citrate synthase which reduce the in vivo activity of this enzyme would allow AAT to compete more effectively with citrate synthase, thereby increasing the formation of acetate esters.

**Figure 1:**
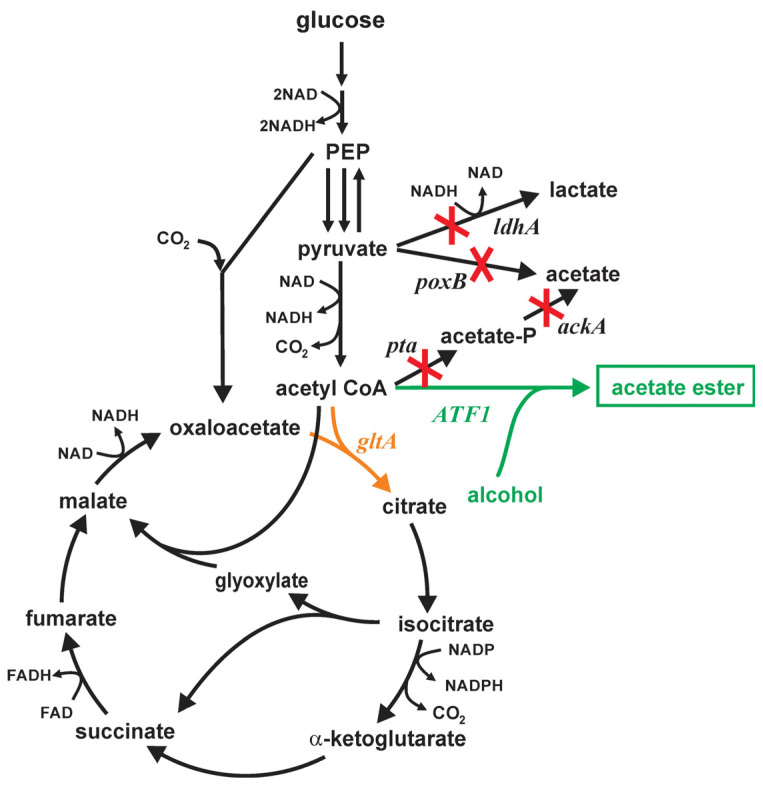
Central carbon metabolism of *E. coli* with acetate ester formation by the expression of the *ATF1*-coding alcohol-O-acetyltransferase from *S. cerevisiae* using exogenously supplied alcohol (green). *E. coli* strains contained knockouts in *ldhA, poxB, pta* and *ackA* genes (red). Citrate synthase activity (orange) was modulated by introducing amino acid substitutions A267T or F383M [21].

This study focuses on the production of several acetate esters in *E. coli* expressing *S. cerevisiae* alcohol-O-acetyltransferase Atf1. Exogenously supplied alcohol serves as the source for the alcohol, while the bacteria are the source for the intracellular acetyl-CoA. In order to minimize the formation of by-products which might reduce acetyl-CoA availability, the strains studied contained knockouts in the *ldhA*, *poxB* and *pta-ackA* genes (Figure 1). To study the effect of diminished citrate synthase activity, strains chromosomally expressing this enzyme with the A267T and F383M substitution were compared.

## 2. Materials and Methods

### 2.1. Strains

Strains used in this study are listed in Table 1. Gene knockout strains were constructed using lambda-red recombination [24], as previously described [23], and selected on Lysogeny Broth (LB) plates supplemented with kanamycin. The kan^R^ marker was removed by expression of FLP recombinase from pCP20 [25]. A homologous recombination method was used to integrate point-mutated *gltA* variants into MEC1381 [21,22,23]. Knockouts were confirmed by PCR, and point-mutated *gltA* genes were amplified from the chromosome, gel purified and sequenced to confirm correct mutations (ACGT, Inc., Wheeling, IL, USA).

### 2.2. Plasmid Construction

The pTrc99A-*ATF1* plasmid was constructed using NEBuilder HiFi Assembly (New England Biolabs, Ipswich, MA, USA). The *ATF1* gene from Saccharomyces cerevisiae was codon optimized (Invitrogen, Thermo Fisher Scientific, Waltham, MA, USA) and cloned into the pTrc99A plasmid [26] to form plasmid pTrc99A-*ATF1*. PrimeStar Max High-Fidelity Polymerase (Takara Bio, Mountain View, CA, USA) was used to amplify DNA for cloning and genome integration. The primers used were CACTGCTGCTGGGTCCGTAAGATCCTCTAGAGTCGACCTG (Trc-HA-ATF-Forward), TTTTCATCGATCTCGTTCATGGTCTGTTTCCTGTGTG (Trc-HA-ATF-Reverse), TTTCACACAGGAAACAGACCATGAACGAGATCGATG (ATF-HA-Trc-Forward) and CAGGTCGACTCTAGAGGATCTTACGGACCCAGCAGCAGTG (ATF-HA-Trc-Reverse). Quick-DNA Miniprep and Zyppy Plasmid Miniprep Kits were used to purify genomic and plasmid DNA (Zymo Research, Irvine, CA, USA). DNA Clean and Concentrator and Zymoclean Gel DNA Recovery Kits were used to purify PCR fragments (Zymo Research, Irvine, CA, USA). The plasmid was confirmed by restriction digest (New England Biolabs, Ipswich, MA, USA) and sequencing (ACGT, Inc., Wheeling, IL, USA).

### 2.3. Media

Shake flask experiments were conducted in 125 mL flasks containing 25 mL defined medium in 50 mM MOPS buffer (pH 7.0) composed of (final concentration, per liter) 8 g glucose, 3.50 g NH_4_Cl, 0.288 g KH_2_PO_4_, 0.502 g K_2_HPO_4_·3H_2_O, 2 g K_2_SO_4_, 20 mg Na_2_(EDTA)·2H_2_O, 0.45 g MgSO_4_·7H_2_O, 0.25 mg ZnSO_4_·7H_2_O, 0.125 mg CuCl_2_·2H_2_O, 1.25 mg MnSO_4_·H_2_O, 0.875 mg CoCl_2_·6H_2_O, 0.06 mg H_3_BO_3_, 0.25 mg Na_2_MoO_4_·2H_2_O, 5.5 mg FeSO_4_·7H_2_O, 20 mg citric acid and 20 mg thiamine·HCl.

Batch experiments were conducted in 2.5 L bioreactors containing 1.25 L defined medium in 25 mM MOPS composed of (final concentration, per liter) 20 g glucose, 8 g NH_4_Cl, 1.2 g KH_2_PO_4_, 2 g K_2_HPO_4_·3H_2_O, 2 g K_2_SO_4_, 20 mg Na_2_(EDTA)·2H_2_O, 0.6 g MgSO_4_·7H_2_O, 0.25 mg ZnSO_4_·7H_2_O, 0.125 mg CuCl_2_·2H_2_O, 1.25 mg MnSO_4_·H_2_O, 0.875 mg CoCl_2_·6H_2_O, 0.06 mg H_3_BO_3_, 0.25 mg Na_2_MoO_4_·2H_2_O, 5.5 mg FeSO_4_·7H_2_O, 50 mg citric acid and 20 mg thiamine·HCl. Shake flasks and bioreactors were supplemented with 150 mg/L ampicillin, as well as alcohols and casamino acids as indicated.

### 2.4. Shake Flask Experiments

A single colony from a Lysogeny Broth (LB) plate was used to inoculate 4 mL LB. After 12–16 h, this culture was transferred to a baffled 125 mL shake flask with 25 mL LB to an initial OD of 0.1. After 6–8 h, this culture was transferred to a baffled 125 mL shake flask with 25 mL defined medium to an initial OD of 0.1. After 6–8 h, this culture (at an OD of 3–5) was transferred to three baffled 125 mL shake flasks with 25 mL defined medium to an initial OD of 0.05 containing 5 g/L of either ethanol, propanol, 1-butanol, 2-butanol or isobutanol. Cultures were grown on a rotary shaker at 250 rpm and 30 °C or 37 °C, and 200 μM IPTG was added to the culture after 2 h of growth. Samples were withdrawn at the beginning and at 8–12 h.

### 2.5. Bioreactor Experiments

A single colony from an LB plate was used to inoculate 4 mL defined medium with 20 g/L glucose. After 12–16 h, this culture was transferred to a baffled 125 mL shake flask containing 25 mL defined medium with 20 g/L of glucose to an initial OD of 0.1. When this culture reached an OD of 4–5, it was transferred to a 2.5 L bioreactor (Bioflo 2000, New Brunswick Scientific Co., New Brunswick, NJ, USA) containing 1.25 L defined medium with 2 g/L casamino acids. After 2 h of growth, 250 mL of isopropyl myristate (as extractant), 8 g propanol and 200 μM IPTG was added to the culture.

Batch studies were conducted in duplicate with an agitation of 400 rpm at 30 °C. Air was supplemented with oxygen at 1.25 L/min to maintain a dissolved oxygen concentration above 40% of saturation. The pH was controlled at 7.0 using 30% (*w*/*v*) KOH. Antifoam 204 (Sigma Chemical Co., St. Louis, MO, USA) was used as necessary to control foaming.

To capture any loss of volatile propyl acetate during bioreactor experiments, the effluent gas was collected in a series of three 250 mL gas washing bottles submerged in ice water. The reported propyl acetate and propanol (masses) are the sum of the chemical in the bioreactor and these three water receivers.

### 2.6. Analytical Methods

The optical density at 600 nm (OD) (UV-650 spectrophotometer, Beckman Instruments, San Jose, CA, USA) was used to monitor cell growth. Samples were routinely frozen at −20 °C for further analysis, and thawed samples were centrifuged (4 °C, 10,000× *g* for 10 min) and filtered (0.45 µm nylon, Acrodisc, Pall Corporation, Port Washington, NY, USA). High-performance liquid chromatography (HPLC) was used to measure sugars and organic acids using RI detection [27].

Gas chromatography (GC) was used to quantify acetate esters and propanol in the bioreactor studies. Samples collected were extracted with 1:1 (*v*/*v*) isopropyl myristate for 24 h. Standard curves were made with the same extractant. A capillary column (Restek, Crossbond 5% diphenyl/95% dimethyl polysiloxane, L = 30 m, ID = 0.32 mm) was used with nitrogen (20 mL/min). The oven temperature was held at 30 °C for 2 min, increased to 210 °C at 15 °C/min, then held at 210 °C for 10 min. The detector was FID, and the injection volume was 5 μL. Student’s *t*-test was applied for comparison of experiments using *p* < 0.05 as the criterion for significance.

## 3. Results

### 3.1. Ester Production

**Figure 2:**
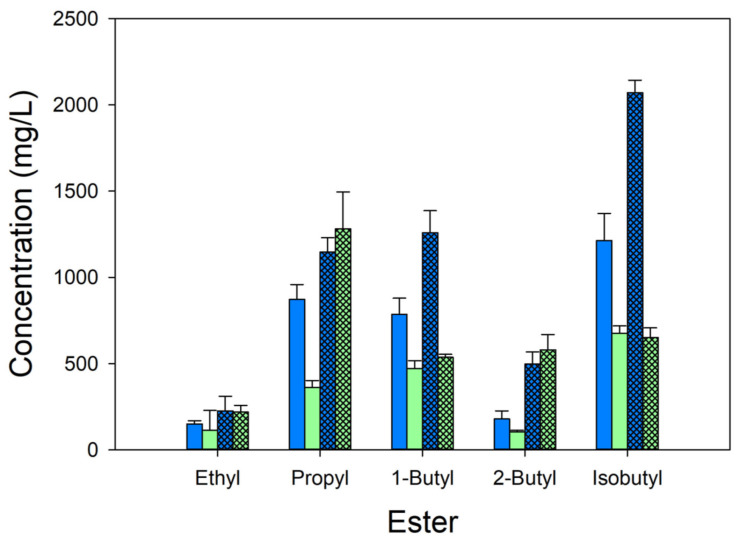
Effect of casamino acids and temperature on acetate ester formation by W Δ*ldhA* Δ*poxB* Δ*pta-ackA* using 8 g/L glucose in shake flask cultures. Cultures were grown at 30 °C (blue) or 37 °C (green) without casamino acids (plain) or supplemented with 2 g/L casamino acids (hatched). Triplicate cultures were grown on a rotary shaker at 250 rpm, and contained 5 g/L alcohol as indicated and 150 mg/L ampicillin. Samples were withdrawn at 8–12 h. All strains contained the pTrc99A-*ATF1* plasmid.

The goal of this work was to use *E. coli* expressing the *ATF1* allele to produce acetate esters. *E. coli* Δ*ldhA* Δ*poxB* Δ*pta-ackA* (MEC1365) containing knockouts of the genes coding for lactate dehydrogenase, pyruvate oxidase, phosphotransacetylase and acetate kinase was transformed with the IPTG inducible plasmid pTrc99A-*ATF1*. We first examined the effects of temperature (30 °C versus 37 °C) and supplementing the medium with casamino acids (none versus 2 g/L) on the otherwise defined medium containing 8 g/L glucose and 5 g/L of one of five alcohols. Several samples were taken at 8–12 h, corresponding to the time the glucose was depleted. The results are summarized in Figure 2.

The conditions resulted in a wide range of ester concentrations depending on the alcohol, the medium or the temperature. Although no single set of conditions led to the greatest ester titer consistently for all five of the acetate esters (Figure 2), some trends were observed. Across both temperatures, in the presence of casamino acids, the addition of ethanol generated significantly lower amounts of the corresponding ester than other alcohols. In the absence of casamino acids, ethanol and 2-butyl acetate both generated significantly lower acetate esters than the other alcohols. Generally, the presence of casamino acids in the medium increased ester titer at any given temperature. (The only exceptions were for 1-butyl and isobutyl alcohols at 37 °C, which showed no significant difference.) The lower temperature (30 °C) generally favored ester formation, particularly for cultures without casamino acids. In contrast, acetate formation was generally favored at the higher temperature. For example, during the formation of ethyl acetate, the acetate yield on glucose was 0.09 ± 0.00 g/g at 30 °C and 0.135 ± 0.01 g/g at 37 °C. Similarly, during the formation of propyl acetate, the acetate yield on glucose was 0.09 ± 0.01 g/g at 30 °C and 0.11 ± 0.01 g/g at 37 °C. Interestingly, no acetate was observed during the formation of butyl acetate at either temperature. Furthermore, no pyruvate was observed, except during the formation of isobutyl acetate, in which case more pyruvate accumulated than acetate (achieving a yield of 0.08 ± 0.02 at 30 °C and 0.13 ± 0.03 g/g at 37 °C). Based on these results, all subsequent experiments used 2 g/L casamino acids and were conducted at 30 °C.

### 3.2. Citrate Synthase Variants

We next focused on three esters, propyl, 1-butyl and isobutyl, to examine the effect of substitutions in citrate synthase (GltA). We selected these esters because the yield of those esters was the greatest in the shake flask studies (Figure 2). We compared ester formation using strains carrying the wild-type citrate synthase (MEC1381) and two strains with modifications in the chromosomally expressed citrate synthase GltA[A267T] or GltA[F383M] (MEC1394 and MEC1410, respectively). Strains were grown at 30 °C using 2 g/L casamino acids as a supplement.

**Figure 3:**
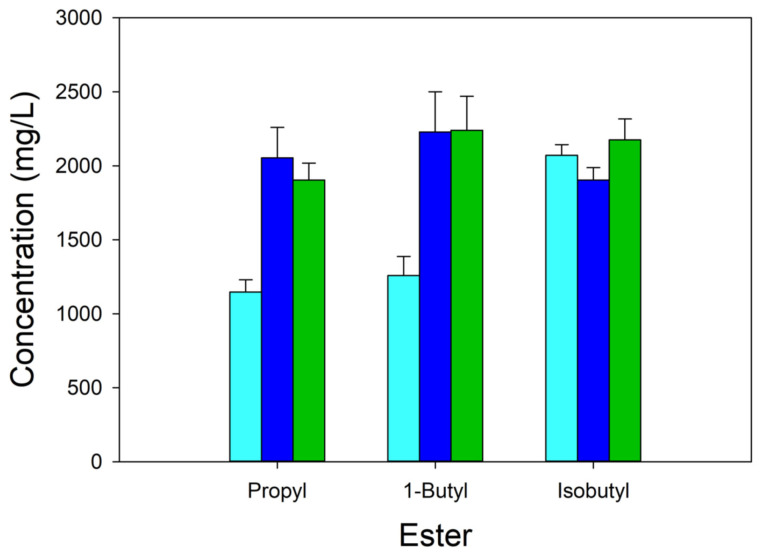
Acetate ester titer by W Δ*ldhA* Δ*poxB* Δ*pta-ackA* strains after growth using 8 g/L glucose in shake flask cultures. Cultures were grown at 30 °C with 2 g/L added casamino acids. Strains expressed different citrate synthase enzymes: wild-type citrate synthase (light blue), A267T (dark blue), F383M (dark green). All strains contained the pTrc99A-*ATF1* plasmid.

For propyl acetate and butyl acetate, the GltA variant strains (MEC1394 and MEC1410) produced greater ester titers compared to the wild-type GltA (MEC1365) strain (Figure 3). For the production of propyl acetate, MEC1394 (2100 ± 200 mg/L) and MEC1410 (1900 ± 100 mg/L) accumulated 70–90% more (*p* < 0.05) propyl acetate compared to the wild-type GltA strain MEC1365 (1100 ± 80 mg/L). There was not a significant difference in the amount of propyl acetate accumulated by the two variant strains MEC1394 (GltA[A267T]) and MEC1410 (GltA[F383M]). For the production of 1-butyl acetate, the same trend was apparent. GltA variant strains MEC1394 (2200 ± 300 mg/L) and MEC1410 (2200 ± 200 mg/L) produced a significantly greater concentration of butyl acetate ester (*p* < 0.05) than the wild-type GltA strain MEC1365 (1300 ± 100 mg/L). As observed for propyl acetate production, no significant difference was observed between the two variant strains. For isobutyl acetate, there was no significant difference in ester concentration between the variants and the wild-type GltA strain.

A greater yield of acetate was generated during the formation of propyl acetate than during the formation of the other two esters. Also, the GltA variants accumulated more acetate than MEC1365. The formation of 1-butyl acetate was accompanied by no acetate and no pyruvate formation for any strain. For example, MEC1365 generated 0.09 ± 0.01 g/g acetate, while MEC1394 (GltA[A267T]) generated 0.13 ± 0.01 g/g and MEC1410 (GltA[F383M]) generated 0.13 ± 0.00 g/g acetate. Pyruvate accumulated only during isobutyl acetate formation, with the GltA variants generating less pyruvate than the wild-type strain (0.03 g/g versus 0.08 g/g).

### 3.3. Effect of Casamino Acids

Many researchers use complex medium components such as protein hydrolysate or yeast extract for the growth of *E. coli* containing a plasmid used to express proteins in an introduced pathway to accumulate a desired product. For example, LB (containing 10 g/L tryptone and 5 g/L yeast extract) supplemented with glucose [9] or terrific broth (12 g/L tryptone, 24 g/L yeast extract and 4 mL/L glycerol) supplemented with glucose [28] has been used for ester production. Also common for ester formation are glucose-containing media supplemented solely with 5–20 g/L yeast extract [8,10,11,29]. In addition to increasing the effective concentration of carbon/energy sources, these complex media provide cells with building block molecules such as amino acids and lipids which greatly facilitates the overexpression of proteins from a plasmid [30,31]. However, the cost of tryptone and yeast extract likely precludes its industrial use in cultures for the production of inexpensive commodity chemicals [32].

**Figure 4:**
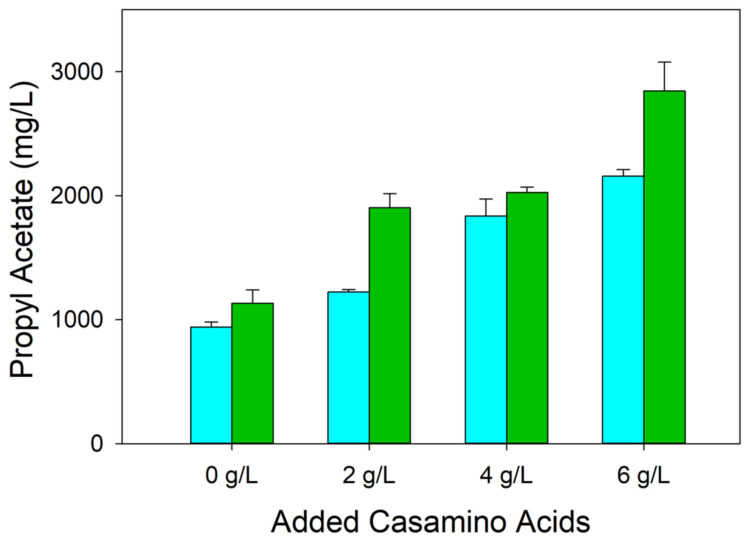
Acetate ester titer by W Δ*ldhA* Δ*poxB* Δ*pta-ackA* expressing wild-type citrate synthase (light blue) and W Δ*ldhA* Δ*poxB* Δ*pta-ackA* expressing F383M variant (dark green) after growth using 8 g/L glucose in shake flask cultures. Cultures were grown at 30 °C with indicated concentration of added casamino acids. All strains contained the pTrc99A-*ATF1* plasmid.

Our positive results using a medium with 2 g/L casamino acids compared to a medium without casamino acids (Figure 2) further demonstrate the benefit of using a protein hydrolysate in a growth and production medium. We were interested to learn if additional casamino acids would further increase ester formation. Also, we wanted to determine whether the improvement in ester titer shown by the use of a citrate synthase variant (Figure 3) was maintained for different concentrations of casamino acids. We therefore compared a range of casamino acid concentrations (0–6 g/L) and compared propyl acetate formation in MEC1365 (wild-type citrate synthase) and MEC1410 (GltA[F383M]), each expressing pTrc99A-*ATF1* (Figure 4). For MEC1365, the propyl acetate titer was greater in each experiment with higher casamino acid concentration (*p* < 0.05). For MEC1410, the propyl acetate titer also increased with increasing casamino acid concentration, except for the 2 g/L and 4 g/L concentrations, which were not significantly different. Moreover, in each case except cultures with 4 g/L casamino acids, the citrate synthase variant (GltA[F383M]) yielded more ester than the strain expressing the wild-type citrate synthase. The 4 g/L casamino acid culture showed no significant difference (*p* > 0.05) between these two strains (Figure 4). These results clearly show the benefit of increased protein hydrolysate for ester production. For the industrial production of esters, the associated increased cost will have to be weighed against this increased yield.

### 3.4. Ester Production in Batch Processes

We next selected propanol to study ester formation in a controlled bioreactor at 30 °C using 20 g/L glucose and 2 g/L casamino acids. Because a bioreactor is aerated by gas sparging, in contrast to flasks shaken on an orbital platform, we anticipated that a significant amount of the volatile ester would be stripped from the bioreactor as previously observed [12]. Therefore, we bubbled the off-gas into three sequential water receivers. Two hours after the commencement of cell growth, 8 g of propanol was introduced into the culture, and the expression of Atf1 was induced with 200 μM IPTG. At this same time (2 h), an organic phase extractant, isopropyl myristate, was introduced into the bioreactor to further capture propyl acetate.

**Figure 5:**
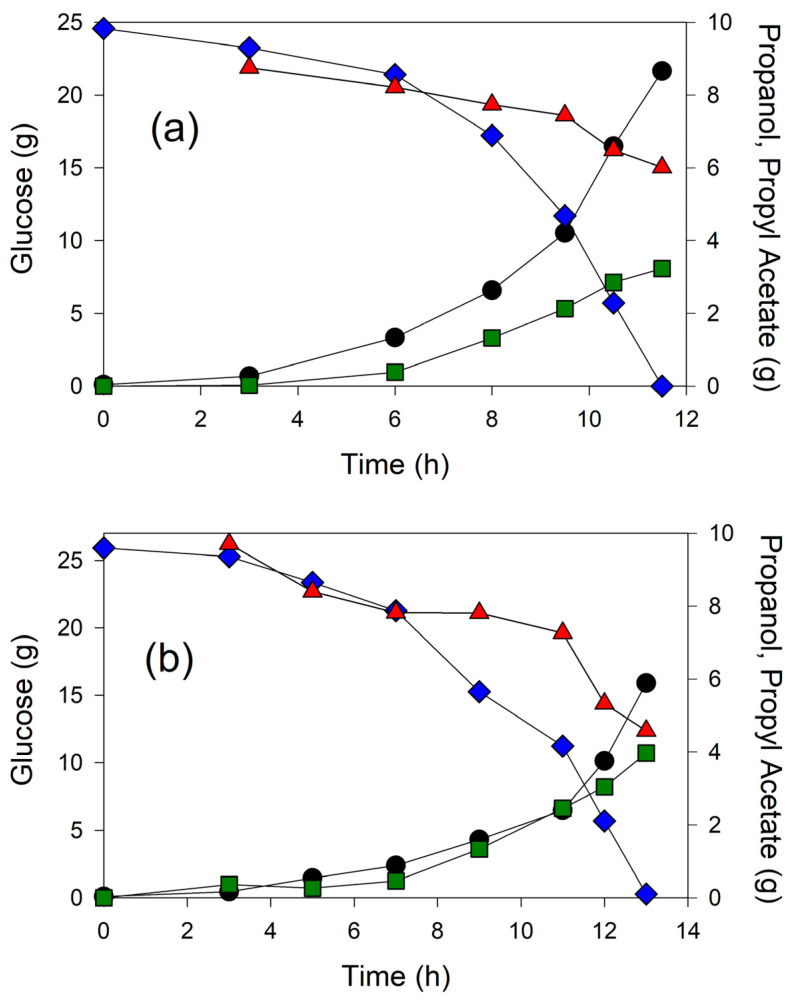
Time course of 1.25 L batch cultivations of (**a**) W Δ*ldhA* Δ*poxB* Δ*pta-ackA* expressing wild-type citrate synthase and (**b**) W Δ*ldhA* Δ*poxB* Δ*pta-ackA* expressing A267T variant. OD (●), Glucose (♦), Propanol (▲), Propyl Acetate (■). All strains contained the pTrc99A-*ATF1* plasmid.

Two strains were compared in the bioreactor for this experiment: MEC1365 (wild-type GltA) and MEC1394 (GltA[A267T]), each expressing pTrc99A-*ATF1*, and Figure 5 shows example results of these batch experiments. MEC1365 expressing pTrc99A-*ATF1* generated 3.2 g propyl acetate from 2.7 g propanol and 24.6 g consumed glucose (Figure 5, effective concentration of 19.7 g/L glucose), corresponding to a mean molar yield of propyl acetate from propanol of 69%. By the end of the process (11–12 h), approximately 16% of the propyl acetate was found in the water receivers, 34% in the bioreactor aqueous phase and 50% in the bioreactor extractant phase. Some propanol was also removed from the bioreactor, with approximately 11% captured in the water receivers and 2% in the bioreactor extractant phase by the end of the process. MEC1394 expressing pTrc99A-*ATF1* generated 3.9 g propyl acetate from 3.5 g propanol and 24.6 g consumed glucose, corresponding to a mean molar yield of propyl acetate from propanol 64%. Similar to the process using MEC1365, the batch studies using MEC1394 showed 18% of the ester in the water receivers, 29% in the bioreactor aqueous phase and 53% in the bioreactor extractant phase by the end of the process (13 h). In the process using MEC1394, 18% of the propanol was in the water receivers and 2% in the bioreactor extractant phase. Thus, the vast majority of propyl acetate appeared in the extractant or water traps (>80%), while the vast majority of the propanol remained in the bioreactor aqueous phase (>80%). Interestingly, because of the volatility of the ester and its partition into the extractant, the maximum propyl acetate present in the bioreactor aqueous phase was always less than 1.0 g/L, even though well over 3 g of ester was generated during each of the 1.25 L processes. The overall yield of propyl acetate from glucose was 0.131 g/g for the strain expressing the wild-type GltA, while a propyl acetate yield of 0.155 g/g was obtained in the strain expressing the GltA[A267T] variant, a 18% increase in ester formation due to the use of a citrate synthase variant.

## 4. Discussion

Small- to medium-chain-length volatile esters have extensive applications in the flavor, fragrance, cosmetic, solvent, paint and coating industries, and high titer and yield are paramount for the biochemical production of esters. In this study, we explored the effect of restricting the flow of carbon at the acetyl-CoA node on the production of acetate esters. This restriction of flux was achieved by knocking out genes, leading to formation of by-products lactate (*ldhA*) and acetate (*pta-ackA* and *poxB*), and by introducing substitutions into citrate synthase, the key entry point to the tricarboxylic acid (TCA) cycle. The substitutions have been previously shown to reduce the flow of acetyl-CoA into the TCA cycle and thereby allow more acetyl-CoA to be available for the formation of products derived from acetyl-CoA [21,22]. Both single amino acid substitutions decrease citrate synthase significantly. Wild-type citrate synthase shows a k_CAT_ of 81 s^−1^ and a K_M_(acetyl-CoA) of 120 μM [33], while GltA[A267T] has a k_CAT_ of 2.1 s^−1^ and a K_M_(acetyl-CoA) of 351 μM [21]. Thus, the GltA[A267T] has a >40-fold lower efficiency than the native citrate synthase at all concentrations of acetyl-CoA. Kinetic parameters for the GltA[F383M] variant were not measured, although the GltA[F383A] variant has a k_CAT_ of 2.7 s^−1^ and a K_M_(acetyl-CoA) of 2000 μM [33]. Moreover, the GltA[F383A] variant shows distinctly sigmoidal behavior [33]. Thus, both variants were selected because they were expected to reduce the citrate synthase activity > 95%, facilitating greater flux of acetyl-CoA towards ester formation.

A low concentration of ethyl acetate was generated under all conditions compared to any of the other esters (Figure 2). This result is consistent with previous studies which have shown that Atf1 has a lower affinity with ethanol than other longer-chain alcohols. Although Atf1 produces ethyl acetate, it does so to a lesser extent than Atf2, another AAT found in yeast [10]. Thus, a much greater concentration of ethanol is required to generate the ester. For example, 200 mM ethanol was shown to generate less ester than 15 mM butanol or 15 mM isoamyl alcohol [34]. Under anaerobic conditions in which citrate synthase is inhibited by accumulation of NADH, 3.8 g/L ethyl acetate is generated in an *E. coli* strain expressing the Eat1 gene from *Wickerhamomyces anomalus* [35].

Of the several esters studied, 2-butyl acetate also accumulated to a comparatively lower titer than other esters regardless of the strain (Figure 2). The low formation of 2-butyl acetate may be due to the toxicity of 2-butanol. A previous study examining the toxicity of various alcohols, acids and esters in *E. coli* growth showed that butanol is among the most inhibitory alcohols [36]. The presence of 4 g/L 2-butanol also negatively affected the growth rate of *S. cerevisiae*, *E. coli* and *Bacillus subtilis*, while 16 g/L butanol inhibited growth and biochemical formation in solventogenic Clostridia [5]. Inexplicably, we observed no difference in isobutyl acetate in the GltA variants compared to the strain expressing the wild-type citrate synthase (Figure 3). It is possible that isobutanol or its acetate ester uniquely affects the acetyl-CoA pool or citrate synthase activity. Clearly, given the results with ethanol, 2-butanol and isobutanol, the benefits derived from the use of citrate synthase variants are alcohol specific.

Cultures grown in medium at 30 °C consistently accumulated greater titers of acetate esters than cultures grown at 37 °C. In another study focused on the production of isoamyl acetate, researchers studied the process at 25 °C, 30 °C and 37 °C and found the greatest ester formation at 25 °C [37]. These authors speculated that the lower temperatures (25 °C and 30 °C) led to more ester formation because the Atf1 enzyme is most active at temperatures close to the optimal temperature for *S. cerevisiae* growth (28 °C–33 °C), and/or protein expression via the Lac operon happens most efficiently at 30 °C. A lower temperature would also affect the degree to which propyl acetate is volatilized from the culture. Interestingly, the formation of acetate differed depending on the temperature and the specific alcohol. Since *poxB* and *pta-ackA* genes were knocked out, the formation of acetate likely occurred because of the presence of thioesterases [23]. That a lower temperature favored ester formation while a higher temperature favored acetate formation suggests merely that the competing enzymes (i.e., introduced Atf1 versus a native thioesterase) had different activities at the two examined temperatures. It remains unclear why acetate formation differed between different alcohol–ester sets. One possibility is that the alcohols (or esters) affect specific enzymes differently, for example, inhibiting a native pathway which leads to less acetate formation. Additional studies are needed to understand the effect of different alcohols on acetate (and ester) formation.

The addition of casamino acids had a positive effect on ester accumulation. These results are consistent with previous studies that showed the addition of a nitrogen source increased microbial acetate ester production [38]. Of course, addition of casamino acids also increases the carbon content of the medium. Availability of building blocks directly from the medium could also simply allow greater availability of acetyl CoA, from which the acetate esters are derived. Casamino acids are acid-hydrolyzed casein, and 2 g/L leads to 1.7 mM glutamate and 1.2 mM proline, the amino acids present in the greatest concentrations [39]. Casamino acids also contain trace amounts of unsaturated fatty acids such as oleic acid, as well as most of the amino acids. Because a wide range of protein hydrolysates and concentrations is used in the development of media and reported in the literature, care should be taken when comparing ester formation by different strains.

Acetate esters are volatile biochemical products, and, in this study, about one-sixth of the product propyl acetate had been removed from the bioreactor by the end of the process (12–14 h), and over 50% of the ester was found in the organic phase. It is likely a prolonged or continuous process would result in greater product removal from the culture phase. Advantages of volatile products include minimization of product inhibition, and potential for in-process product recovery. Of additional interest would be novel approaches to increase the volatilization, such as the use of reduced pressure, higher temperature or greater gas transfer.

### GltA Variants

*E. coli* containing a deletion in the *gltA* gene cannot grow on glucose as the sole carbon source without glutamate (e.g., found in casamino acids) or other metabolizable carbon sources to supply the TCA cycle. Thus, a dilemma exists in processes to generate biochemical products derived from acetyl-CoA. The lower the activity of citrate synthase, the greater the potential for high production but the lower the growth rate. Although not examined in this study, a Δ*gltA* strain expressing Atf1 could be grown on a protein hydrolysate, and then, when the culture becomes depleted of nutrients like glutamate, the process switched from growth to an ester formation phase in the presence of glucose only. An analogous concept using a genetic circuit for modulating the activity of citrate synthase employs a “conditional knockout”, whereby a metabolic toggle in *Escherichia coli* can inhibit the expression of a specific gene [40]. This approach has been applied to isopropanol formation in conditionally expressed *gltA*, resulting in three-fold improved titer and over three-fold improved yield [40].

Another strategy, which could be used in combination with nutrient or genetic circuit approaches, involves the modification of chromosomally expressed citrate synthase. Our results show that GltA variant strains containing the A267T (MEC1394) or F383M (MEC1410) substitutions accumulated significantly greater titers of propyl and 1-butyl acetate compared to the wild-type GltA strain. In a controlled bioreactor, we observed a 18% increase in yield simply by modifying citrate synthase, supporting the hypothesis that GltA substitutions would redirect acetyl-CoA from acetate to the ester.

## Figures and Tables

**Table 1:** List of strains used in this study.

Designation	Genotype
ATCC 9637	Wild-type *E. coli* W
MEC1365	*E. coli* W Δ*ldhA* Δ*poxB* Δ*pta-ackA*
MEC1380	*E. coli* W Δ*ldhA* Δ*poxB* Δ*pta-ackA* Δ*gltA*::Kan
MEC1381	*E. coli* W Δ*ldhA* Δ*poxB* Δ*pta-ackA* Δ*gltA*
MEC1394	*E. coli* W Δ*ldhA* Δ*poxB* Δ*pta-ackA* Δ*gltA*::*gltA*^[A267T]^
MEC1410	*E. coli* W Δ*ldhA* Δ*poxB* Δ*pta-ackA* Δ*gltA*::*gltA*^[F383M]^

## Data Availability

All data are available by directing queries to corresponding author.
